# Anti-Inflammatory Effects of Thymoquinone in Atherosclerosis: A Mini Review

**DOI:** 10.3389/fphar.2021.758929

**Published:** 2021-12-15

**Authors:** Xin-Fang Leong, Ker Woon Choy, Aspalilah Alias

**Affiliations:** ^1^ Department of Craniofacial Diagnostics and Biosciences, Faculty of Dentistry, Universiti Kebangsaan Malaysia, Kuala Lumpur, Malaysia; ^2^ Department of Anatomy, Faculty of Medicine, Universiti Teknologi MARA, Selangor, Malaysia; ^3^ Department of Basic Sciences and Oral Biology, Faculty of Dentistry, Universiti Sains Islam Malaysia, Kuala Lumpur, Malaysia

**Keywords:** atherosclerosis, inflammation, thymoquinone, nuclear factor-kappa B, mitogen-activated protein kinase

## Abstract

Atherosclerosis poses serious health problems and increases the risk of various cardiovascular diseases, including myocardial infarction, heart failure, ischemic stroke, and peripheral arterial disease. Atherosclerosis patients require long-term medications to prevent complications, some of which are costly and may result in unwanted adverse reactions. Natural products have emerged as potential sources of bioactive compounds that provide health benefits in cardiovascular diseases. Increased inflammation and vascular remodeling have been associated with atherosclerosis pathogenesis. The molecules involved in signaling pathways are considered valuable targets for new treatment approaches. Therefore, this review aimed to summarize the available evidence of the anti-inflammatory effects of thymoquinone, the major active compound isolated from *Nigella sativa* L*.*, *via* inflammatory signaling pathways in atherosclerosis. Specifically, nuclear factor-κB and mitogen-activated protein kinase signaling pathways were considered. Furthermore, the potential toxic effects elicited by thymoquinone were addressed. These findings suggest a potential role of thymoquinone in managing atherosclerosis, and further studies are required to ascertain its effectiveness and safety profile.

## Introduction

Atherosclerosis is a major cause of cardiovascular disease (CVD) worldwide, including myocardial infarction, heart failure, ischemic stroke, and peripheral arterial disease. According to the Global Burden of Cardiovascular Diseases and Risk Factors (Roth et al., 2020), CVD prevalence has increased from 271 million to 523 million from 1990 to 2019. The CVD mortality had a relative increase of 6.5% in 2019, reaching 18.6 million deaths. It is estimated that 23.6 million people globally will die from CVDs by 2030 (WHO, 2013). The rising burden of CVDs on individuals and the healthcare system warrants research on atherosclerotic diseases and implementation of preventive measures.

There are several theories on atherosclerosis pathogenesis, including lipid theory, oxidative theory, response to injury theory, and inflammatory theory (Minelli et al., 2020). Various inflammatory cells and inflammatory mediators are responsible for fatty streak formation, progression, and rupture of atheromatous plaques (Libby, 2021). The major signaling pathways that mediate inflammation include nuclear factor-κB (NF-κB) and mitogen-activated protein kinase (MAPK). Hence, modulating these inflammatory signaling pathways to produce anti-inflammatory actions may serve as potential therapeutic targets for atherosclerosis management.

There has been increasing interest in medicinal herbs or plants for the treatment and prevention of various diseases, including atherosclerosis. Plant-based traditional medicines have attracted considerable attention owing to their availability, cost, safety, and efficacy. The World Health Organization (WHO) reported that approximately 60–80% of the population use traditional medicines or herbal remedies for their primary health care, particularly in developing countries. It is recommended that the WHO Traditional Medicine Strategy 2014–2023 is implemented for national traditional medicine programs. This strategy aims to explore the potential use of traditional medicine for health and wellness, in addition to encouraging its safe and effective use (Zhang, 2018).

*Nigella sativa* L., also known as black seed or black cumin, is a plant traditionally used for medicinal purposes in the Middle East, India, Northern Africa, and Europe. *N. sativa* L. has been used to treat various ailments, including asthma, hypertension, diabetes, inflammation, cough, headache, eczema, fever, and dizziness (Salehi et al., 2021). *N. sativa* L. is a flowering plant belonging to the family Ranunculaceae. The fruit contains angular-shaped black seeds, which are regarded as the most important component in view of their beneficial health effects (Tavakkoli et al., 2017).

*N. sativa* L. contains various bioactive compounds, including thymoquinone (TQ), dithymoquinone, thymol, and thymohydroquinone. Among the isolated compounds, TQ was the most abundant. Hence, the extensive therapeutic benefits exerted by *N. sativa* L. may be attributed to TQ (Alagawany et al., 2021). Previous studies have shown that TQ possesses various pharmacological properties, including antioxidant (Abd-Elkareem et al., 2021), antimicrobial (Mouwakeh et al., 2018), antihypertensive (Enayatfard et al., 2018), antidiabetic (Bule et al., 2020), lipid-lowering (Majdalawieh et al., 2021), neuroprotective (Abulfadl et al., 2018), gastroprotective (Bukar et al., 2017), anticancer (Edris, 2021), and anti-inflammatory (Alkharfy et al., 2018; Ahmad et al., 2020). Given the potential health benefits of TQ, the present study aimed to examine the available evidence on its anti-inflammatory effects in atherosclerosis *via* signaling pathway modulation, and to highlight its potential toxicity.

## Inflammatory Signaling Pathways

### NF-κB Pathway

NF-κB pathway activation is regulated by inhibitory proteins of the κB family (IκB) kinase through IκB phosphorylation (Christian et al., 2016), which causes its degradation by the proteasome, leading to the release of NF-κB for nuclear translocation and gene transcription activation. This pathway regulates inflammatory cytokine production and inflammatory cell recruitment, which contribute to the inflammatory response.

### MAPK Pathway

MAPKs consist of three members: extracellular signal-regulated kinases (ERKs), p38 MAPK, and c-Jun N-terminal kinases (JNKs). ERKs are generally activated by mitogens and differentiation signals (Sun et al., 2015), while p38 MAPK and JNK are activated by inflammatory stimuli and stress (Chan et al., 2017). MAPK activation leads to phosphorylation and activation of transcription factors, which are responsible for inflammatory response regulation (Chen et al., 2018).

## Atheroprotective Effects of TQ *via* Modulation of Signaling Pathways

**FIGURE 1:**
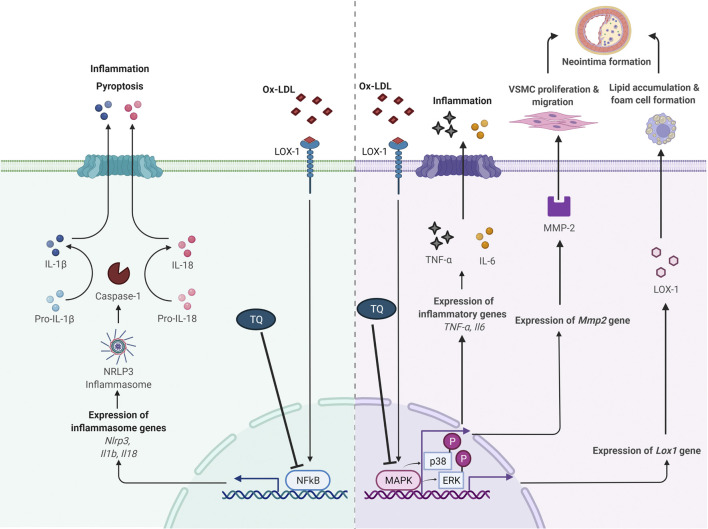
Proposed antiatherogenic effects of thymoquinone in atherosclerosis via modulation of NF-κB and MAPK signaling pathways. IL, interleukin; LOX-1, lectin-like oxidized low-density lipoprotein receptor 1; MAPK, mitogen-activated protein kinase; MMP-2, matrix metalloproteinase 2; NF-κB, nuclear factor κB; ox-LDL, oxidized low-density lipoprotein; NLRP3, NOD-like receptor protein 3; p-ERK, phosphorylation of extracellular signal-regulated kinase; p-p38, phosphorylation of p38; TNF-α, tumor necrosis factor alpha; VSMC, vascular smooth muscle cell; ⊥, suppress. Adapted from “Suppression of Inflammasome by IRF4 and IRF8 is Critical for T Cell Priming”, by BioRender.com (2021). Retrieved from https://app.biorender.com/biorender-templates

Studies involving signaling pathways have documented that cytokine-mediated inflammation is a crucial element in atherosclerosis pathogenesis. Hence, inflammatory response regulation is a fundamental aspect in atherosclerosis prevention and treatment (Liu et al., 2017; Sun et al., 2018). The proposed atheroprotective effects of TQ *via* NF-κB and MAPK pathway modulation are shown in Figure 1.

### Effect of TQ in NF-κB Pathway

Vascular cell adhesion molecule 1, intercellular adhesion molecule 1, chemokines interleukin 8 (IL-8), and monocyte chemoattractant protein 1 (MCP-1) are major molecules that recruit circulating mononuclear leukocytes to the arterial intima. This process is important in atherosclerosis and is mediated by NF-κB activation (Mussbacher et al., 2019). Amartey et al. (2019) reported that concurrent treatment with TQ (6.25 μg/ml) showed a tendency to reduce inflammatory response by suppressing IL-6 and IL-8 protein levels in human vascular endothelial cells (HVECs) exposed to lipopolysaccharides (LPS, 100 ng/ml) at 24 h. Furthermore, TQ downregulated the mRNA expression of important inflammation regulators vascular endothelial growth factor (VEGF) and MCP-1 in LPS-treated cells. VEGF mediates angiogenesis, whereas MCP-1 is involved in endothelial monocyte activation.

Furthermore, the expression of NOD-like receptor protein 3 (NLRP3) inflammasome and IL-1β was attenuated by TQ in HVECs exposed to LPS for 24 h. In the presence of inflammation, ten-eleven translocation 2 (TET-2) gene expression increased with concurrent administration of TQ (Amartey et al., 2019). The role of TET-2 in atherosclerosis has been elucidated by Fuster et al. (2017). Macrophages with TET-2 deficiency led to increased pro-inflammatory cytokine IL-1β secretion, which is dependent on the action of NLRP3 (Grebe et al., 2018). According to these findings, TQ plays a regulatory role in inflammation and monocyte recruitment, and modulates NLRP3 and TET-2 *in vitro*. However, no positive controls were used in this study. Media-treated cells were used as a control to differentiate the effects of LPS and TQ. Further studies on multiple cell lines and *in vivo* studies are required to confirm the anti-inflammatory effect of TQ against atherosclerosis. This study did not investigate the mechanism of action of TQ. The anti-inflammatory action of TQ could be due to NF-κB suppression in view of its regulatory role in NRLP3 and pro-inflammatory cytokines such as the IL-1 family (Liu et al., 2017).

Hyperlipidemia has been reported to accelerate lipid accumulation, atherosclerosis, and chronic inflammation in apolipoprotein E knockout (ApoE^−/-^) or low-density lipoprotein receptor-deficient (LDL-R^−/−^) mice (Zhao et al., 2018). ApoE^−/-^ and LDL-R^−/−^ mice are two models commonly used in atherosclerosis research that require hypercholesterolemia induction. Their mechanisms of enhancing atherosclerosis development and the involved lipoproteins are different (Getz and Reardon, 2016). ApoE deficiency in macrophages may contribute to hypercholesterolemia, while the lack of LDL-R in hepatocytes is responsible for hypercholesterolemia in ApoE^−/-^ and LDL-R^−/−^ models. The following studies utilized normal diet-fed mice as the control group.

Xu et al. (2018) reported that concurrent treatment with TQ (oral, 25 mg/kg/day for 8 weeks) decreased serum high-sensitivity C-reactive protein levels in high-cholesterol diet-fed adult male ApoE^−/-^ mice. Additionally, TQ suppressed the upregulation of tumor necrosis factor α (TNF-α) and IL-6 expression in cardiac tissues isolated from high-cholesterol diet-fed mice. Similar results were reported by Pei et al. (2020) in LDL-R^−/−^ mice. Pei et al. (2020) documented that a high-cholesterol diet supplemented with TQ (oral, 50 mg/kg/day for 8 weeks) reduced TNF-α and IL-6 serum levels and gene expression in mice. Cluster of differentiation 68 markers, which are highly expressed in macrophages, were reduced following TQ administration, indicating a reduction in macrophage numbers in the cardiac tissue of high-cholesterol diet-fed LDL-R^−/−^ mice. In addition, TQ administration downregulated the increased protein and gene expression of NLRP3, caspase-1, IL-1β, and IL-18 induced by a high-cholesterol diet. Decreased NF-κB protein expression was observed following concurrent high-cholesterol diet with TQ supplementation in LDL-R^−/−^ mice (Pei et al., 2020). Pyroptosis, a programmed cell death mechanism mediated by NLRP3 activation, has been associated with hyperlipidemia development. NLRP3 activation stimulates caspase-1, an IL-1 converting enzyme that cleaves precursors of the inflammatory cytokines IL-1β and IL-18. Subsequently, the release of pro-inflammatory cytokines is enhanced, leading to pyroptosis (Borges et al., 2017).

### Effect of TQ in MAPK Pathway

Oxidized low-density lipoprotein (ox-LDL) contributes to atherosclerosis-associated inflammation (Rhoads and Major, 2018). Ox-LDL causes endothelial dysfunction, leading to adhesion molecule expression and monocyte recruitment in the subendothelial space. Ox-LDL is taken up by macrophages *via* lectin-like ox-LDL receptor 1 (LOX-1). LOX-1 expression is upregulated by ox-LDL (Barreto et al., 2021). Additionally, ox-LDL promotes the growth and migration of smooth muscle cells, monocytes, and macrophages (Pirillo et al., 2013).

Xu et al. (2018) revealed that ApoE^−/-^ mice receiving a high-cholesterol diet concurrent with TQ (oral, 25 mg/kg/day) for 8 weeks had reduced LOX-1 protein and gene expression in cardiac tissues. Lipid deposition, foam cell formation, and ERK phosphorylation (p-ERK) are regulated by protein kinases (Lin et al., 2012). Upregulated LOX-1 expression was suppressed by ERK inhibitors, suggesting that MAPK pathway activation is a crucial signaling event in LOX-1 gene regulation (Zhang et al., 2017). p-ERK was significantly reduced in ApoE^−/-^ mice receiving TQ and a high-cholesterol diet than in mice without TQ supplementation (Xu et al., 2018). Therefore, TQ may regulate LOX-1 *via* the p-ERK pathway. ERK inhibition may exert potential antiatherosclerotic effects, as indicated by reduced uptake of ox-LDL and foam cell formation in hypercholesteremic TQ-supplemented ApoE^−/-^ mice (Xu et al., 2018).

Pei et al. (2020) investigated the effect of TQ on hyperlipidemia-induced cardiac damage in male LDL-R^−/−^mice. It was demonstrated that concurrent treatment with TQ (oral, 50 mg/kg/day) reduced total cholesterol and LDL-cholesterol levels in addition to the pro-inflammatory cytokines in mice fed a high-cholesterol diet for 8 weeks. There was a reduction in lipid accumulation and inflammatory cell infiltration in the cardiac tissue of TQ-administered mice compared to that in the non-supplemented mice. TQ decreased p38 and p-ERK levels in high-cholesterol diet-fed mice. These findings suggest that TQ suppresses high-cholesterol diet-induced inflammation and cardiac damage *via* p38 and ERK pathway inhibition.

Various pathological events are involved in vascular remodeling in response to vascular damage, including endothelial dysfunction, vascular smooth muscle cell (VSMC) proliferation and migration, arterial calcification, and extracellular matrix remodeling (Wang and Khalid, 2018; Zhang et al., 2021). Such injury-induced vascular remodeling is primarily due to excessive proliferation and migration of VSMCs and medial VSMC invasion into the intimal space, eventually leading to neointimal formation.

Zhu et al. (2019) reported that TQ (10, 12.5, 15 μmol/L) suppressed platelet-derived growth factor (PDGF, 40 ng/ml)-induced VSMC proliferation at 24 h. Furthermore, TQ decreased α-smooth muscle actin and Ki-67-positive cells, confirming the antiproliferative effect of TQ on VSMCs. Additionally, TQ (5–15 μmol/L) attenuated PDGF-stimulated VSMC migration, and TQ (15 μmol/L) blocked the activity and expression of matrix metalloproteinase 2 (MMP-2) in VSMCs at 24 h (Zhu et al., 2019). MMP-2 is involved in VSMC migration *via* extracellular matrix degradation (Xiao et al., 2018). Inhibition of p38 activation also blocked MMP-2 expression (Zhu et al., 2019). Hence, p38 might be responsible for the inhibitory effect of TQ on MMP-2 expression. TQ treatment increased the number of apoptotic VSMCs in the presence of reactive oxygen species (Zhu et al., 2019). The results showed that TQ abolished the upregulation of B-cell lymphoma 2 (Bcl-2), cleaved caspase 3, and cleaved poly (ADP-ribose) polymerase, and blocked Bcl-2-associated X protein (Bax) downregulation. It has been suggested that the pro-apoptotic effect of TQ is mediated *via* the mitochondria-dependent apoptosis pathway. Zhu et al. (2019) also documented that 8 mg/kg and 16 mg/kg TQ stopped the increase in neointimal area and neointima/media ratio, and attenuated neointimal formation in atherosclerosis at 14 days using a rat carotid artery ligation model. Therefore, the inhibitory activity of TQ on VSMC proliferation and migration may be attributed to the blockade of p38 MAPK activation.

## Potential Toxicity of TQ

### Acute and Subacute Toxicity

A single intraperitoneal (i.p.) dose of TQ was administered to BALB/c mice at doses ranging from 10 to 80 mg/kg body weight to test the oxidative effect of TQ after 24 h (Table 1). TQ at 40 and 80 mg/kg caused a considerable increase in malondialdehyde levels and catalase activity in the kidneys and liver (Harzallah et al., 2012). Oral acute toxicity of TQ from doses 0.5–3 g/kg was evaluated in male Swiss albino mice (Badary et al., 1998). Death occurred within the first 3 h associated with hypoactivity and respiratory problems, particularly with 2 or 3 g/kg TQ. No mortality was reported until 24 h. There was an increase of plasma activity of alanine aminotransferase, lactate dehydrogenase, creatinine phosphokinase, and increased plasma concentrations of urea and creatinine with 2 or 3 g/kg TQ. Besides, a reduction of reduced glutathione levels was reported (Table 1).

**TABLE 1:** Toxicity profile of TQ.

Toxicity test	Dosage of TQ per kg body weight	Type of animal	Frequency/Route of administration	Observation time	Findings	References
**Acute and Subacute**	10, 20, 40, 80 mg/kg	BALB/c mice	Single/Intraperitoneal	24 h	- No change in body, liver, and kidney weights	Harzallah et al. (2012)
					- Increased tissue MDA and CAT levels at 40 or 80 mg/kg TQ	
	0.5, 1, 2, 3 g/kg	Male Swiss albino mice	Single/Oral	24 h	- LD_50_ was 2.4 g/kg	Badary et al. (1998)
					- Increased plasma concentrations of urea, creatinine, ALT, LDH, CPK and reduced GSH levels in liver, kidney and heart at 2 or 3 g/kg TQ	
	50, 75, 100, 125, 150 mg/kg	Male and female Albino mice	Single/Intraperitoneal	24 h	- Abdominal muscle spasms and ataxia, worsened with higher doses.	Al-Ali et al. (2008)
	25, 50, 75, 100, 150 mg/kg	Male and female Wistar rats			- LD_50_ values 10–15 times greater than TQ dose for anti-inflammatory, antioxidant, or anticancer effects	
	250, 500, 1,000, 1,500, 2000 mg/kg	Male and female Albino mice	Single/Oral		- Drowsy and dyspneic over time before dying or recovering	
	100, 500, 1,000, 1,500, 2000 mg/kg	Male and female Wistar rats			- LD_50_ values 100–150 times greater than TQ dose for beneficial effect	
	20, 30, and 40 mg/kg 200, 300, and 500 mg/kg	Male and female Wistar rats	Single/Intraperitoneal Single/Oral	24 h intervals for 5 days	- Loss of body weight, acute pancreatitis and elevation of serum amylase level	Abukhader (2012)
					Short term sign of toxicity (i.e., loss of body weight, mild abdominal distention, and dyspnea)	
					- 500 mg/kg TQ caused two fatalities due to complication from bowel obstruction	
	TQNLC or TQ (5, 50, and 300 mg/kg)TQNLC or TQ (1, 10,100 mg/kg)	Female BALB/c miceMale and femaleBALB/c mice	Single/Oral Daily/Oral	14 days 28 days	- No weight loss	Ong et al. (2016)
					- No abnormal behavior	
					- Mild hepatotoxicity	
					- NOAEL of TQNLC and TQ was 10 mg/kg/d for mice in both sexes	
	TQNLC (25 mg/kg)	Female Sprague- Dawley rats	Single/Intravenous	14 days	- Normal body weight, hematological, biochemical and histopathological profile	Yazan et al. (2019)
					- Inflammation at site of injection	
	TQRFNE at 20 ml/kg (containing 44.5 mg TQ)	Male and female Sprague-Dawley rats	Single/Oral	14 days	- Normal body weight gains and hematological profile	Tubesha et al. (2013)
					- Normal key enzymes of the liver and kidney, levels of urea and creatinine as well as liver histopathological examination	
**Subchronic**	30, 60, 90 mg/kg	Male Swiss albino mice	Daily/Oral	90 days	- Normal plasma concentrations of urea, creatinine, triglycerides, ALT, LDH, and CPK	Badary et al. (1998)
					- Normal liver, kidneys and heart histopathological examination	
**Teratogenic**	15, 35, 50 mg/kg	Pregnant Wistar rats	Single injection on gestation day 11 or 14/Intraperitoneal	On gestation day 18	- No effects on fetus when 35 mg/kg TQ was given on day 14 of gestation	AbuKhader et al. (2013)
					- Increased serum amylase level, acute pancreatitis, organ adhesion and steatonecrosis at 35 or 50 mg/kg TQ on day 11 of gestation	
	10, 40, 80 mg/kg	Pregnant Wistar rats	Daily for 7 days, gestation week 2 or 3/Oral	Postnatal day 14 and 21	- 40 mg/kg TQ reduced body weight of offspring while 80 mg/kg TQ led to pregnancy loss when treated at gestation week 2	Abdollahzade Fard et al. (2021)
					- 40 or 80 mg/kg TQ caused a lower birth weight but increased body weight on postnatal days 14 and 21 when treated at gestation week 3	
					- 80 mg/kg TQ caused 50% reduction in the size of the litter when treated at gestation week 3	

ALT, alanine aminotransferase; CAT, catalase; CPK, creatinine phosphokinase; GSH, reduced glutathione; LD50, median lethal dose; LDH, lactate dehydrogenase; MDA, malondialdehyde; NOAEL, no observed adverse effect level; TQ, thymoquinone; TQNLC, TQ in a nanostructured lipid carrier; TQRFNE, TQ-rich fraction nano-emulsion.

Al-Ali et al. (2008) showed that the LD_50_ values for TQ in albino mice were 104.7 and 870.9 mg/kg after i. p. and oral administration, respectively. Furthermore, LD_50_ values for i. p. injection and oral ingestion of TQ in Wistar rats were recorded as 57.5 and 794.3 mg/kg, respectively. Abukhader (2012) revealed that the maximum tolerated doses (MTDs) for i. p. TQ injection were 22.5 and 15 mg/kg in male and female rats, respectively, whereas the MTD for oral TQ was 250 mg/kg in both male and female rats. Thus, TQ is regarded as a reasonably safe drug, particularly when administered orally.

Acute toxicity was compared between encapsulated TQ in a nanostructured lipid carrier (TQNLC) and TQ in female BALB/c mice (Ong et al., 2016). Mice administered with 300 mg/kg TQ died within 24 h. In contrast, a mouse treated with 300 mg/kg TQNLC died after 24 h. In the subacute toxicity study (Ong et al., 2016), oral administration of 100 mg/kg TQNLC or TQ for 28 days did not cause mortality in either male or female mice.

A single injection of 25 mg/kg TQNLC was administered to the tail of female Sprague-Dawley rats (Yazan et al., 2019). The same dose was administered to the other rats at 48 h intervals. Intravenous administration of 25 mg/kg TQNLC did not induce toxicity in rats during the 14-days observation period. Male and female Sprague-Dawley rats were observed for 14 days after receiving a single dose of TQ-rich fraction nano-emulsion at 20 ml/kg (Tubesha et al., 2013). The animals appeared normal and healthy throughout the study (Table 1).

In summary, the route of administration can influence the severity of TQ-induced toxicity. Oral administration has a better safety profile than i. p. injections. Compared to that of TQ alone, the use of TQ together with nanostructured lipid carriers or nano-emulsions has less evidence of toxicity, suggesting their potential use during TQ administration.

### Subchronic Toxicity

Male Swiss albino mice were administered 30, 60, or 90 mg/kg TQ for 90 days *via* drinking water (Badary et al., 1998). No signs of toxicity were noted (Table 1).

### Teratogenicity

Decreased maternal body weight and complete fetal resorption were reported after a single i. p. injection of 35 mg/kg or 50 mg/kg TQ to pregnant rats on day 11 of gestation (Abukhader et al., 2013). Administration of 50 mg/kg TQ on day 14 resulted in a higher incidence of fetal resorption, and viable fetuses did not show malformations (Table 1). Complete pregnancy loss was reported in pregnant Wistar rats administered 80 mg/kg TQ orally at the second gestational week for 7 days (Abdollahzade Fard et al., 2021). Reduced offspring body weight was recorded on postnatal days 14 and 21 by TQ (oral, 40 mg/kg). However, pregnant rats treated with TQ at gestation week 3 did not show such toxicity. In conclusion, i. p. injection of TQ between 35 mg/kg and 50 mg/kg during gestation has exhibited teratogenicity, suggesting that doses lower than 35 mg/kg could be safer to avoid fetal abnormalities or deformities. Moreover, failed pregnancy is associated with TQ administered orally at 80 mg/kg and at gestation week 2. Therefore, prenatal TQ administration should be carefully assessed.

## Conclusion and Future Perspectives

Although *N. sativa* L. has long been used for treating diseases and enhancing general health, research into its therapeutic potential and mechanisms of action has just begun. Metabolomics is a useful technology for analyzing the chemical composition of *N. sativa* L. to allow its authentication and to ensure uniformity in bioactivity for quality control (Farag et al., 2021). Limited studies have investigated the anti-inflammatory effects of TQ in atherosclerosis. No positive controls were used in the available published studies. The comparative anti-inflammatory effects of TQ cannot be appreciated. Hence, future studies should incorporate positive controls to validate the effectiveness of TQ as an anti-inflammatory agent. Previous studies have indicated the possible involvement of the NF-κB and MAPK pathways in mediating the anti-inflammatory effects of TQ. However, its direct involvement in such signaling pathways requires exploration. Further investigation is warranted to identify the associated pathways and to determine the molecular targets that mediate the protective effects of TQ in atherosclerosis.

TQ has been shown to be toxic *in vitro* and *in vivo* studies, indicating the requirement for more in-depth research to provide a more complete toxicological profile for TQ before considering this promising natural product as a therapeutic agent for human use. The TQ dosage required to achieve optimal anti-inflammatory benefits in humans remains unknown and requires further investigation. Moreover, the protective effects of TQ have yet to be verified in clinical trials, and more safety assessments are needed to determine the potential toxicities of TQ for long-term use in humans. Therefore, more research is required to confirm its traditional use as a therapy for atherosclerosis.

## References

[B1] Abd-Elkareem M. Abd El-Rahman M. A. M. Khalil N. S. A. Amer A. S. (2021). Antioxidant and Cytoprotective Effects of *Nigella Sativa* L. Seeds on the Testis of Monosodium Glutamate Challenged Rats. Sci. Rep. 11, 13519. 10.1038/s41598-021-92977-4 34188150 PMC8242002

[B2] Abdollahzade Fard A. Saboory E. Tahmazi Y. Rasmi Y. Dindarian S. Parsamanesh N. (2021). Effect of Orally-Administrated Thymoquinone during Pregnancy on Litter Size, Pentylenetetrazol-Induced Seizure, and Body Weight in Rat Offspring. Iran J. Basic Med. Sci. 24, 30–37. 10.22038/ijbms.2020.47479.10930 33643567 PMC7894635

[B3] Abukhader M. M. Khater S. H. Al-Matubsi H. Y. (2013). Acute Effects of Thymoquinone on the Pregnant Rat and Embryo-Fetal Development. Drug Chem. Toxicol. 36, 27–34. 10.3109/01480545.2011.648326 22360537

[B4] Abukhader M. M. (2012). The Effect of Route of Administration in Thymoquinone Toxicity in Male and Female Rats. Indian J. Pharm. Sci. 74, 195–200. 10.4103/0250-474X.106060 23440704 PMC3574528

[B5] Abulfadl Y. S. El-Maraghy N. N. Ahmed A. A. E. Nofal S. Badary O. A. (2018). Protective Effects of Thymoquinone on D-Galactose and Aluminum Chloride Induced Neurotoxicity in Rats: Biochemical, Histological and Behavioral Changes. Neurol. Res. 40, 324–333. 10.1080/01616412.2018.1441776 29464986

[B6] Ahmad A. Alkharfy K. M. Jan B. L. Ahad A. Ansari M. A. Al-Jenoobi F. I. (2020). Thymoquinone Treatment Modulates the Nrf2/HO-1 Signaling Pathway and Abrogates the Inflammatory Response in an Animal Model of Lung Fibrosis. Exp. Lung Res. 46, 53–63. 10.1080/01902148.2020.1726529 32053036

[B7] Al-Ali A. Alkhawajah A. A. Randhawa M. A. Shaikh N. A. (2008). Oral and Intraperitoneal LD_50_ of Thymoquinone, an Active Principle of *Nigella Sativa*, in Mice and Rats. J. Ayub. Med. Coll. Abbottabad. 20, 25–27. 19385451

[B8] Alagawany M. Elnesr S. S. Farag M. R. Abd El-Hack M. E. Khafaga A. F. Sharun K. (2021). ““Health-Promoting Activities of Nigella Sativa Essential Oil,” in Black Cumin (Nigella Sativa),” in Seeds: Chemistry, Technology, Functionality, and Applications. Food Bioactive Ingredients. Editor Fawzy Ramadan M. (Cham: Springer). 10.1007/978-3-030-48798-0_29

[B9] Alkharfy K. M. Ahmad A. Jan B. L. Raish M. (2018). Thymoquinone Reduces Mortality and Suppresses Early Acute Inflammatory Markers of Sepsis in a Mouse Model. Biomed. Pharmacother. 98, 801–805. 10.1016/j.biopha.2018.01.028 29571249

[B10] Amartey J. Gapper S. Hussein N. Morris K. Withycombe C. E. (2019). *Nigella Sativa* Extract and Thymoquinone Regulate Inflammatory Cytokine and TET-2 Expression in Endothelial Cells. Artery Res. 25, 157–163. 10.2991/artres.k.191114.002

[B11] Badary O. A. Al-Shabanah O. A. Nagi M. N. Al-Bekairi A. M. Elmazar M. M. A. (1998). Acute and Subchronic Toxicity of Thymoquinone in Mice. Drug Dev. Res. 44, 56–61. 10.1002/(sici)1098-2299(199806/07)44:2/3<56:aid-ddr2>3.0.co;2-9

[B12] Barreto J. Karathanasis S. K. Remaley A. Sposito A. C. (2021). Role of LOX-1 (Lectin-like Oxidized Low-Density Lipoprotein Receptor 1) as a Cardiovascular Risk Predictor: Mechanistic Insight and Potential Clinical Use. Arterioscler. Thromb. Vasc. Biol. 41, 153–166. 10.1161/ATVBAHA.120.315421 33176449 PMC9186447

[B13] Borges P. V. Moret K. H. Raghavendra N. M. Maramaldo Costa T. E. Monteiro A. P. Carneiro A. B. (2017). Protective Effect of Gedunin on TLR-Mediated Inflammation by Modulation of Inflammasome Activation and Cytokine Production: Evidence of a Multitarget Compound. Pharmacol. Res. 115, 65–77. 10.1016/j.phrs.2016.09.015 27641928

[B14] Bukar M. A. Ishaya H. B. Dibal N. I. Attah M. O. (2017). Gastroprotective Effect of *Nigella Sativa* Seed on Ethanol-Induced Gastric Ulcer in Rats. Libyan J. Med. Sci. 1, 63–67. 10.4103/LJMS.LJMS_23_17

[B15] Bule M. Nikfar S. Amini M. Abdollahi M. (2020). The Antidiabetic Effect of Thymoquinone: A Systematic Review and Meta-Analysis of Animal Studies. Food Res. Int. 127, 108736. 10.1016/j.foodres.2019.108736 31882078

[B16] Chan L. P. Liu C. Chiang F. Y. Wang L. F. Lee K. W. Chen W. T. (2017). IL-8 Promotes Inflammatory Mediators and Stimulates Activation of P38 MAPK/ERK-NF-κB Pathway and Reduction of JNK in HNSCC. Oncotarget 8, 56375–56388. 10.18632/oncotarget.16914 28915597 PMC5593568

[B17] Chen L. Deng H. Cui H. Fang J. Zuo Z. Deng J. (2018). Inflammatory Responses and Inflammation-Associated Diseases in Organs. Oncotarget 9, 7204–7218. 10.18632/oncotarget.23208 29467962 PMC5805548

[B18] Christian F. Smith E. L. Carmody R. J. (2016). The Regulation of NF-Κb Subunits by Phosphorylation. Cells 5, 12. 10.3390/cells5010012 PMC4810097 26999213

[B19] Edris A. E. (2021). in “Thymoquinone: Chemistry and Functionality,” in Black Cumin (Nigella Sativa) Seeds: Chemistry, Technology, Functionality, and Applications. Food Bioactive Ingredients. Editor Fawzy Ramadan M. (Cham: Springer). 10.1007/978-3-030-48798-0_8

[B20] Enayatfard L. Mohebbati R. Niazmand S. Hosseini M. Shafei M. N. (2018). The Standardized Extract of *Nigella Sativa* and its Major Ingredient, Thymoquinone, Ameliorates Angiotensin II-Induced Hypertension in Rats. J. Basic Clin. Physiol. Pharmacol. 30, 51–58. 10.1515/jbcpp-2018-0074 30269105

[B21] Farag M. A. Saad H. H. Hegazi N. M. (2021). “Rediscovering Nigella Seeds Bioactives Chemical Composition Using Metabolomics Technologies,” in ” in Black Cumin (Nigella Sativa) Seeds: Chemistry, Technology, Functionality, and Applications. Food Bioactive Ingredients. Editor Fawzy Ramadan M. (Cham: Springer). 10.1007/978-3-030-48798-0_10

[B22] Fuster J. J. MacLauchlan S. Zuriaga M. A. Polackal M. N. Ostriker A. C. Chakraborty R. (2017). Clonal Hematopoiesis Associated with TET2 Deficiency Accelerates Atherosclerosis Development in Mice. Science 355, 842–847. 10.1126/science.aag1381 28104796 PMC5542057

[B23] Getz G. S. Reardon C. A. (2016). Do the Apoe-/- and Ldlr-/- Mice Yield the Same Insight on Atherogenesis? Arterioscler. Thromb. Vasc. Biol. 36, 1734–1741. 10.1161/ATVBAHA.116.306874 27386935 PMC5001905

[B24] Grebe A. Hoss F. Latz E. (2018). NLRP3 Inflammasome and the IL-1 Pathway in Atherosclerosis. Circ. Res. 122, 1722–1740. 10.1161/CIRCRESAHA.118.311362 29880500

[B25] Harzallah H. J. Rjiba K. Maaloul A. Abid-Essafi S. Mahjoub T. (2012). Oxidative and Genotoxic Effects of Thymoquinone, Nigella Sativa Active compoundBalb/c Mice. Afr. J. Food Sci. 6, 529–534. 10.5897/AJFS12.06610.5897/ajmr11.1073

[B26] Libby P. (2021). Inflammation in Atherosclerosis-No Longer a Theory. Clin. Chem. 67, 131–142. 10.1093/clinchem/hvaa275 33393629

[B27] Lin C. S. Lin F. Y. Ho L. J. Tsai C. S. Cheng S. M. Wu W. L. (2012). PKCδ Signalling Regulates SR-A and CD36 Expression and Foam Cell Formation. Cardiovasc. Res. 95, 346–355. 10.1093/cvr/cvs189 22687273

[B28] Liu T. Zhang L. Joo D. Sun S. C. (2017). NF-κB Signaling in Inflammation. Signal. Transduct. Target. Ther. 2, 17023. 10.1038/sigtrans.2017.23 29158945 PMC5661633

[B29] Majdalawieh A. F. Yousef S. A. Abu-Yosef I. A. (2021). Thymoquinone, a Major Constituent in *Nigella Sativa* Seeds, Is a Potential Preventative and Treatment Option for Atherosclerosis. Eur. J. Pharmacol. 909, 174420. 10.1016/j.ejphar.2021.174420 34391767

[B30] Minelli S. Minelli P. Montinari M. R. (2020). Reflections on Atherosclerosis: Lesson from the Past and Future Research Directions. J. Multidiscip. Healthc. 13, 621–633. 10.2147/JMDH.S254016 32801729 PMC7398886

[B31] Mouwakeh A. Telbisz Á. Spengler G. Mohácsi-Farkas C. Kiskó G. (2018). Antibacterial and Resistance Modifying Activities of Nigella Sativa Essential Oil and its Active Compounds against. Listeria Monocytogenes 32, 737–743. 10.21873/invivo.11230210.21873/invivo.11302 PMC6117775 29936453

[B32] Mussbacher M. Salzmann M. Brostjan C. Hoesel B. Schoergenhofer C. Datler H. (2019). Cell Type-specific Roles of NF-Κb Linking Inflammation and Thrombosis. Front. Immunol. 10, 85. 10.3389/fimmu.2019.00085 30778349 PMC6369217

[B33] Ong Y. S. Saiful Yazan L. Ng W. K. Noordin M. M. Sapuan S. Foo J. B. (2016). Acute and Subacute Toxicity Profiles of Thymoquinone-Loaded Nanostructured Lipid Carrier in BALB/c Mice. Int. J. Nanomedicine 11, 5905–5915. 10.2147/IJN.S114205 27877037 PMC5108596

[B34] Pei Z. W. Guo Y. Zhu H. L. Dong M. Zhang Q. Wang F. (2020). Thymoquinone Protects against Hyperlipidemia-Induced Cardiac Damage in Low-Density Lipoprotein Receptor-Deficient (LDL-R^-/-^) Mice via its Anti-inflammatory and Antipyroptotic Effects. Biomed. Res. Int. 2020, 4878704. 10.1155/2020/4878704 33178827 PMC7644313

[B35] Pirillo A. Norata G. D. Catapano A. L. (20132013). LOX-1, OxLDL, and Atherosclerosis. Mediators Inflamm., 152786. 10.1155/2013/152786 PMC3723318 23935243

[B36] Rhoads J. P. Major A. S. (2018). How Oxidized Low-Density Lipoprotein Activates Inflammatory Responses. Crit. Rev. Immunol. 38, 333–342. 10.1615/CritRevImmunol.2018026483 30806246 PMC6527110

[B37] Roth G. A. Mensah G. A. Johnson C. O. Addolorato G. Ammirati E. Baddour L. M. (2020). Global burden of Cardiovascular Diseases and Risk Factors, 1990-2019: Update from the GBD 2019 Study. J. Am. Coll. Cardiol. 76, 2982–3021. 10.1016/j.jacc.2020.11.010 33309175 PMC7755038

[B38] Salehi B. Quispe C. Imran M. Ul-Haq I. Živković J. Abu-Reidah I. M. (2021). Sen S, Nigella Plants - Traditional Uses, Bioactive Phytoconstituents, Preclinical and Clinical Studies. Front. Pharmacol. 12, 625386. 10.3389/fphar.2021.625386 33981219 PMC8107825

[B39] Sun L. F. An D. Q. Niyazi G. L. Ma W. H. Xu Z. W. Xie Y. (2018). Effects of Tianxiangdan Granule Treatment on Atherosclerosis via NF-Κb and P38 MAPK Signaling Pathways. Mol. Med. Rep. 17, 1642–1650. 10.3892/mmr.2017.8067 29257205 PMC5780105

[B40] Sun Y. Liu W. Z. Liu T. Feng X. Yang N. Zhou H. F. (2015). Signaling Pathway of MAPK/ERK in Cell Proliferation, Differentiation, Migration, Senescence and Apoptosis. J. Recept. Signal. Transduct. Res. 35, 600–604. 10.3109/10799893.2015.1030412 26096166

[B41] Tavakkoli A. Mahdian V. Razavi B. M. Hosseinzadeh H. (2017). Review on Clinical Trials of Black Seed (Nigella Sativa) and its Active Constituent, Thymoquinone. J. Pharmacopuncture 20, 179–193. 10.3831/KPI.2017.20.021 30087794 PMC5633670

[B42] Tubesha Z. Imam M. U. Mahmud R. Ismail M. (2013). Study on the Potential Toxicity of a Thymoquinone-Rich Fraction Nanoemulsion in Sprague Dawley Rats. Molecules 18, 7460–7472. 10.3390/molecules18077460 23803717 PMC6270347

[B43] Wang X. Khalil R. A. (2018). Matrix Metalloproteinases, Vascular Remodeling, and Vascular Disease. Adv. Pharmacol. 81, 241–330. 10.1016/bs.apha.2017.08.002 29310800 PMC5765875

[B44] WHO (2013). Global Health Observatory. Geneva, Switzerland: World Health Organization.

[B45] Xiao X. L. Hu N. Zhang X. Z. Jiang M. Chen C. Ma R. (2018). Niclosamide Inhibits Vascular Smooth Muscle Cell Proliferation and Migration and Attenuates Neointimal Hyperplasia in Injured Rat Carotid Arteries. Br. J. Pharmacol. 175, 1707–1718. 10.1111/bph.14182 29486057 PMC5913402

[B46] Xu J. Zhu L. Liu H. Li M. Liu Y. Yang F. (2018). Thymoquinone Reduces Cardiac Damage Caused by Hypercholesterolemia in Apolipoprotein E-Deficient Mice. Lipids Health Dis. 17, 173. 10.1186/s12944-018-0829-y 30049280 PMC6062953

[B47] Yazan L. S. Mohd Azlan S. N. Ansar F. H. Z. Gopalsamy B. (2019). Acute Toxicity Study of Intravenous Administration of Thymoquinone-Loaded Nanostructured Lipid Carrier (TQ-NLC) in Sprague Dawley Rat. Mal. J. Med. Health Sci. 15, 51–57.

[B48] Zhang L. Yao J. Yao Y. Boström K. I. (2021). Contributions of the Endothelium to Vascular Calcification. Front. Cel. Dev. Biol. 9, 620882. 10.3389/fcell.2021.620882 PMC8165270 34079793

[B49] Zhang Q. (2018). Global Situation and WHO Strategy on Traditional Medicine. Tradit. Med. Mod. Med. 1, 11–13. 10.1142/S257590001820001X

[B50] Zhang Z. Zhang D. Du B. Chen Z. (2017). Hyperoside Inhibits the Effects Induced by Oxidized Low-Density Lipoprotein in Vascular Smooth Muscle Cells via oxLDL-LOX-1-ERK Pathway. Mol. Cel. Biochem. 433, 169–176. 10.1007/s11010-017-3025-x PMC5554480 28434118

[B51] Zhao Y. Yang Y. Xing R. Cui X. Xiao Y. Xie L. (2018). Hyperlipidemia Induces Typical Atherosclerosis Development in Ldlr and Apoe Deficient Rats. Atherosclerosis 271, 26–35. 10.1016/j.atherosclerosis.2018.02.015 29459263

[B52] Zhu N. Xiang Y. Zhao X. Cai C. Chen H. Jiang W. (2019). Thymoquinone Suppresses Platelet-Derived Growth Factor-BB-Induced Vascular Smooth Muscle Cell Proliferation, Migration and Neointimal Formation. J. Cel. Mol. Med. 23, 8482–8492. 10.1111/jcmm.14738 PMC6850929 31638340

